# Open Iris - An Open Source Framework for Video-Based Eye-Tracking Research and Development

**DOI:** 10.1101/2024.02.27.582401

**Published:** 2024-03-03

**Authors:** Roksana Sadeghi, Ryan Ressmeyer, Jacob L. Yates, Jorge Otero-Millan

**Affiliations:** Herbert Wertheim School of Optometry and Vision Science, University of California, Berkeley; Bioengineering, University of Washington; Herbert Wertheim School of Optometry and Vision Science, University of California, Berkeley; Herbert Wertheim School of Optometry and Vision Science, University of California, Berkeley

**Keywords:** Software and its engineering, Software notations and tools, Development frameworks and environments, Application specific development environments, Eye-tracking, eye movement, open-source

## Abstract

Eye tracking is an essential research tool in many fields, yet existing solutions often face cost and flexibility limitations for customized applications. We present OpenIris, a user-friendly and adaptable open-source framework developed in C# for video-based eye-tracking. It is designed for diverse research applications capable of tracking eye movements in three dimensions, including torsional eye movements. Its modular design allows easy customization for different hardware systems, tracking, and calibration pipelines. It can be remotely controlled from other devices or programs and can record eye movements online from cameras or post-process recorded videos. Currently implemented binocular pupil tracking pipelines can achieve frame rates of more than 500Hz when running on standard computer workstations. With OpenIris framework, we aim to fill a gap in the research tools available for high precision and high-speed eye tracking, especially in environments that require custom solutions that are not currently well-served by commercial eye trackers.

## 1 INTRODUCTION

Eye tracking technology is used in an ever-growing list of fields. In basic science research, the study of eye movements contributes to a comprehensive understanding of various facets of human brain function, such as vision [7], vestibular processing [5,17], attention [13], motor learning [19], and emotions [27]. In clinical settings, eye tracking systems play a crucial role in medical diagnosis across various disciplines including neurology [15], ophthalmology [8], otology [14], psychology [23], and psychiatry [22]. The field of human-computer interfaces extensively relies on eye trackers to enhance communication between humans and computers/robots [1,6]. Additionally, marketing research harnesses the power of eye tracking systems to objectively evaluate the efficacy of different advertisements or designs to capture and retain attention. [3]

There is a wide variety of eye tracking technologies developed over the last decades. Each with unique features and compromises tailored to specific applications. Some are small and can be head mounted [10], others are large taking up almost entire room [16], some are expensive and high frequency, while others are affordable and low frequency, etc. Eye tracking systems differ significantly in their ability to detect a range of eye movement amplitudes with some of them only being able to measure large eye movements, others only small movements, and very few systems performing well over small and large movements [21]. Recently, video-based eye tracker systems, in particular, have become the most common choice in head mounted or desk mounted configurations because they offer valuable features at a potentially lower cost than other types of eye trackers. A high-quality video-based eye tracker can deliver adequate precision, high-speed real-time tracking, and a wide detection range for eye movements from less than 0.5 degrees to more than 20 degrees. [21] However, options for commercially available video-based eye trackers are limited, expensive and are often closed systems that lack flexibility and customizability for different applications (for example EyeLink ^®^, Tobii, and Eyegaze Inc.). Thus, commercial systems are not suitable for users who require customized hardware configurations or specialized eye-tracking algorithms.

Therefore, many open-source eye tracking systems have appeared as an alternative to the commercial eye trackers which address either the customization or cost-effectiveness needed in research applications. (Table 1) This is possible, in part, because the hardware necessary for video-based eye tracking are few, namely: an infrared (IR) illuminator, a video camera with a lens and an IR filter, and a computer to acquire and process the video. These systems enable the presentation of stimuli in synchrony with the eye tracker system and provide toolboxes for visualizing and analyzing recorded data. Although various open-source eye tracking systems are available, there is still a lack of a comprehensive platform that offers high-performance, customizable features for different applications, along with a user-friendly graphical interface.

This paper introduces an open-source, extendable, performant, and user-friendly video-based eye tracker framework that meets the reliability and speed requirements for research. Our framework, OpenIris, implements many features that are commonly required for eye tracking systems, including multi-threading, data buffering, recording, logging, graphical interfacing, and network communication. It is written in the C# programming language, which allows for low-level optimization resulting in real-time binocular performance exceeding 500 frames per second. However, what separates OpenIris from other solutions is its modular design. By leveraging a plugin-based architecture, OpenIris enables its users to easily develop and deploy custom hardware configurations, tracking pipelines, and calibration routines.

Here we describe the structure and features of OpenIris and explain the options for developing new customizable plugins. We report the performance of the OpenIris with examples for pupil and torsional tracking. OpenIris fills a gap by providing an open-source, interactive, and user-friendly video-based eye tracker that aims to be friendly to the end-user and to developers interested in testing new hardware or new strategies for eye tracking. With its modular programming, high-speed tracking capabilities, customizable features, and remote-control capability, OpenIris empowers researchers to conduct efficient eye-tracking experiments.

## 2 OPENIRIS FEATURES

**Figure 1:**
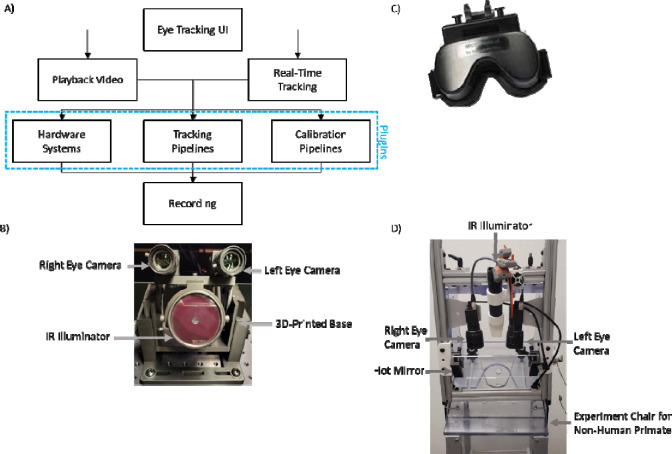
a) Simplified structure of OpenIris program, b) Example video-based eye tracking setup that used in OpenIris desktop system with two cameras and one IR illuminator c) Micromedical head-mounted eye tracking system by Intracoustics [30] and d) setup used for non-human primate studies

OpenIris implements a set of core features that are common to most eye tracking systems, while allowing its functionality to be extended through the development of modular plugins. This architecture allows plugin developers to focus on the more conceptually complex image processing techniques for tracking without the need to implement the often more tedious requirements involving user interface, real time multithreading, data storage, error handling, and configuration. For the end user, OpenIris provides a user-friendly graphical user interface, ensuring ease of use and accessibility. For the programmer, the modular structure of the platform provides the flexibility of building three types of plugins (Figure 1) that can be loaded into OpenIris as independent libraries without the need of rebuilding the entire platform, and it can be interchanged at run time. First, it allows for plugins for different video camera hardware and configurations, accepting adjustable camera features such as frame rate, frame size, orientation, etc. Second, plugins to add or modify different tracking methods for measuring pupil movements, iris rotations, and reflections movements, or even head movements. A third type of plugin is used to modify the calibration method.

### 2.1 Core features

#### 2.1.1 User Interface:

**Figure 2:**
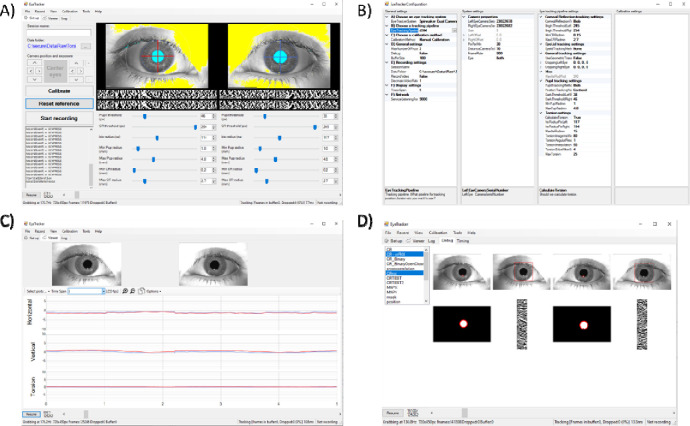
Example print-screen of the OpenIris a) main window setting tab with adjustable parameters, b) configuration window to select the plugins, c) view tab that shows online traces, and d) debug tab that can be used for debugging pipeline performance.

The graphical interface of OpenIris is designed for user-friendly navigation and control. It features a setup tab which displays eye images in real-time, in addition to adjustable settings defined by the tracking pipeline plugin being used. The viewer tab presents eye data traces alongside corresponding videos, offering a comprehensive view of the tracking output. The calibration tab facilitates straightforward monitoring and adjustment of calibration process, with an interface defined by the calibration plugin in use. A log tab is also available to display errors or messages. Finally, a configuration window comprises three columns that allow users to set parameters for OpenIris in general or for each of the three main plugins ( Figure 2).

For advanced users and developers, a debug mode can be enabled which offers an additional tab with a detailed view of tracked objects at different processing stages. This feature aids in debugging the tracking pipeline as well as providing insights into the system’s performance. The plugin developer does not need to implement the user interface elements, they just need to indicate that a particular image of any intermediate processing step should be added to the debug tab.

#### 2.1.2 Remote Control:

In addition to its graphical user interface, OpenIris offers alternative methods of interaction. OpenIris can be utilized purely as a library, providing an API to incorporate it in any other software. Alternatively, OpenIris features network interfaces to remotely communicate with other pieces of software via UDP, TCP, or HTTP protocols. This enables synchronization with other programs written in different languages such as Python or Matlab and running it on different computers/devices. This feature allows remote control of the eye-tracking system, providing flexibility for experiment designs, such as running experiments in complete darkness or implementing gaze contingent displays.

#### 2.1.3 Offline / Batch Analysis:

OpenIris offers offline and batch analysis features, allowing users to reprocess recorded videos with different parameters to refine eye traces. To reprocess multiple videos, the user can adjust the parameters for each video and process all videos in a batch.

#### 2.1.4 Recording:

Data recording includes two primary outputs: a text file containing the results of frame-by-frame tracking, and, optionally, the raw videos obtained from the cameras. The videos are recorded in raw format to allow for their post processing without any loss, which is fundamental in the development and optimization of new tracking methods.

#### 2.1.5 Buffering and Multithreading:

OpenIris natively implements an image buffer which connects the video acquisition and image processing plugins. Due to the variation in processing time caused by non-constant-time algorithms or CPU usage by other processes, some frames may take substantially longer to process than the sampling rate of the eye cameras. OpenIris can often eliminate frame drops – which are caused by these spikes in processing time – by storing up to a set number of unprocessed frames in a first-in-first-out buffer. By reducing the size of this image buffer, it is possible to prioritize real-time latency at the risk of dropping frames.

Additionally, OpenIris runs plugin-defined image processing pipelines in contained threads, which allows for otherwise single-threaded algorithms to leverage multiple processing cores without custom solutions. The number of threads can be easily adjusted by the user using the configuration window.

#### 2.1.6 Configuration:

OpenIris stores all configurations, including system and plugin settings, as a serializable class. This architecture allows new plugins to define settings simply by defining their type and default value as properties in a class which inherits from a base settings class. OpenIris then automatically loads and interprets these settings at run time using reflection, provides a user interface to edit the values of these settings, and saves the settings in a text file. All of these features are handled automatically, eliminating complexity during plugin development.

### 2.2 Plugins

#### 2.2.1 Camera hardware systems

The main function of OpenIris’ hardware system plugins is to establish communication with camera(s) for capturing raw images, which are subsequently processed by later plugins. (Table 2 List of abstract and virtual methods for three main plugins Table 2) OpenIris simplifies the integration of cameras tailored to the users’ specific requirements. Each camera which may be used by the system comes with unique settings associated with its characteristics. Developers have the flexibility to initialize, start, and capture images from their chosen camera(s) by providing specific code snippets. In addition, the design of the OpenIris user interface enables the users to create options for camera(s) settings to be dynamically changed during online tracking.

As an example of a hardware system for a binocular eye tracker, we commonly use two Blackfly S USB3 Flir cameras (with a resolution of 720 × 540 and a frequency of up to 500 frames per second; [31]), each paired with a 50mm focal length lens and an IR filter. A 3D-printed base holds the cameras and an infrared illuminator. (Figure 1b)

Within the OpenIris systems plugin, we take advantage of the camera API to synchronize the capture of the two cameras via hardware connection. In this case, the left-eye camera is assigned as Primary, which sends a signal to the right eye camera to indicate when it should open the shutter and capture a new image. This ensures perfect temporal alignment of the two cameras. Additionally, within the plugin, the user interface was modified to add an option for setting the tracking frequency as a one-time adjustable parameter at the onset of the tracking process before camera initialization. Moreover, the cameras’ gain and region of interest was made adjustable, allowing real-time modifications during tracking.

Figure 1 b, c, and d display some of the different configurations that OpenIris has been adapted for. These include desktop systems with one or two cameras, head mounted goggles, and animal recording rigs.

#### 2.2.2 Tracking pipelines

The main function of tracking pipeline plugins is to process the images and extract relevant features from them. Each camera frame enters the image processing pipeline, which employs the image processing libraries, such as “OpenCV” [32] and its wrapper for C# emguC, for locating and extracting objects of interest. Similar to hardware systems settings, the OpenIris allows developers to add features to the user interface for adjusting image processing settings in real-time during tracking such as brightness thresholds, or minimum and maximum sizes for objects being tracked. (Table 2)

**Figure 3:**
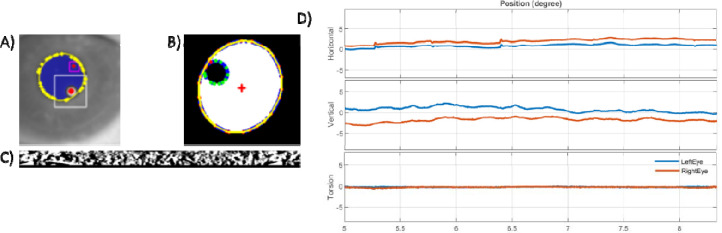
Example of detecting a) p1 and p4 shown in red circles for digital DPI, b) corneal reflection in green and pupil in yellow contour for pupil – CR method, and c) detecting iris pattern for tracking torsional eye movement. d) Example eye traces in degree for left eye in blue and right eye in red color.

For instance, a tracking pipeline is already implemented within OpenIris to locate the pupil in an infrared camera frame using different methods including centroid, convex hull or ellipse fitting. In addition to the pupil tracking, the pipeline includes detecting corneal reflections (also known as glints) for pupil – CR method, and tracks the iris for torsional eye movements. ([18]; Figure 3b,c)

An example of externally developed plugins is provided by the independent contributor (second author), who has developed a dual-Purkinje eye tracking pipeline for OpenIris that detects reflections of the illuminator from the cornea surface and the inner surface of the lens ([24]; Figure 3a).

#### 2.2.3 Calibration pipelines

The main function of calibration pipeline plugins is to establish a mapping between the features extracted by the tracking pipeline and the actual motion of the eye. This mapping can be achieved either through image and geometric analysis or by analyzing behavior in interaction with a display. Initially, raw eye-tracking data is represented in units of pixel on the image. A calibration method translates these pixel values into degrees, corresponding to visual angles.

The calibration pipelines in OpenIris facilitate this conversion process by allowing users to define their own calibration methods. (Table 2) These methods can be based on established eye models or implemented through regression models that correlate known locations with corresponding eye positions. This modular approach empowers users to customize the calibration process to their specific experimental needs, ensuring accurate and meaningful conversion of pixel data to visual angles. The calibration pipeline implemented for behavioral calibration also includes network communication between a computer presenting the targets and a computer executing OpenIris to record the eye positions.

## 3 PERFORMANCE

The overall performance of OpenIris ultimately depends on the specific plugins employed in an experiment. To establish a baseline performance measurement for processing time and data quality, we conducted tests on a simple configuration for binocular pupil tracking utilizing two cameras and one illuminator mentioned above. A participant looked at the central dot for a total of 6 minutes (with 20s intervals and 5s rests). OpenIris recorded the data at 500 Hz and was controlled within a Matlab R2021b program on a PC with processor of i9–11900 at 2.50 GHz, and installed RAM of 64.0 GB.

The study was approved by the University of California, Berkeley Institutions IRB and followed the tenets of the Declaration of Helsinki. The subject signed an informed consent form to participate after being informed of the goals and methods of the study.

### 3.1 Process Time

For images of size 720 × 540 pixels, using maximum five processing threads the baseline average time from a frame arriving at the computer to the completion of data processing, without any image processing, was 0.14 ± 0.18 ms (mean ± standard deviation). Introducing a simple pupil tracking, centroid method, the average processing time was 0.79 ± 0.70 ms.

Using advanced tracking methods for pupil detection, such as Ellipse fitting, the recorded average time was 2.50 ± 1.80 ms, tracking pupil and corneal reflection took 3.70 ± 3.20 ms and tracking pupil and torsional eye movement, 9.70 ± 6.70 ms. The recordings included zero percent dropped frame in all conditions.

### 3.2 Data Quality

The saved video from one session was post-processed at 500 Hz with pupil detection (using ellipse fitting method), pupil and a corneal reflection, and pupil and torsion detection. Figure 3d shows an example horizontal, vertical and torsional eye positions. The root mean square eye velocity in horizontal (h) and vertical (v) directions with pupil were: h 18.55 deg/s, v 40.98 deg/s, with pupil and a corneal reflection: h 47.59 deg/s, v 65.25 deg/s, and the root mean square eye velocity for torsion was: 19.99 deg/s.

## 4 SUMMARY

In conclusion, OpenIris is a user-friendly and adaptable tool for eye-tracking research. Its modular design allows easy customization, and key features like the remote control and batch analysis enhance its usability. OpenIris has the potential to impact diverse research areas, from psychology to human-computer interaction. Looking forward, expanding the tracking algorithm and fostering collaborative development could further enhance its utility. OpenIris stands as a valuable and versatile resource for eye-tracking studies, balancing simplicity with the flexibility needed for various research applications.

## Figures and Tables

**Table 1:** Comparing available open-source eye tracking systems.

Name	Year	Frame Rate (Hz)	Hardware Compatibility	Post-Process	Binocular	GraphicalUser-Interface	Software Language

ITU Gaze Tracker [29]	2010	30	Yes	No	No	Yes	C#
GazeParser [20]	2013	500	Yes	NA	Yes	No	Python
Pupil [12]	2014	22	No	No	Yes	NA	Python and C
Oculomatic[28]	2016	600	Yes	No	No	Yes	C++
openEyeTrack [4]	2019	715	No	NA	NA	NA	C++
DeepVOG [25]	2019	130	No	No	No	No	Python
RemoteEye [9]	2020	500	No	Yes	Yes	Yes	C/C++
EyeLoop [2]	2021	1000	Yes	No	No	Yes	Python
Using DeepLabCut [26]	2021	30	No	No	Yes	NA	Python
A low-cost, high-performance video-based binocular eye tracker for psychophysical research [11]	2021	395	No	No	Yes	NA	C++

**Table 2:** List of abstract and virtual methods for three main plugins

Plugin	Abstract / Virtual Method	Description

Hardware Systems	CreateAndStartCameras	Initializes the cameras to start capturing frames for them.
	CreateVideos	Opens the videos to start capturing frames for them.
	PreProcessImages	Prepares the images after they are grabbed from the videos or cameras and before they are processed by the tracking pipeline. This can be used to split, crop or rotate the images depending on the hardware configuration.
	PostProcessImagesAndData	Receives the images and also the output eye data from the pipeline for any additional post processing or any additional data from the system to append.
	OpenEyeTrackingSystemUI	Opens a custom UI for this eye tracking system.

Tracking Pipelines	Process	Gets the current image and processes it to obtain the eye data.
	UpdatePipelineEyeImage	Updates the image of the eye on the setup tab allowing the pipeline to customize the display of the eye image.
	GetQuickSettingsList	Get the list of tracking settings that will be shown as sliders in the setup UI.

Calibration Pipelines	ProcessForEyeModel	Process the eye data from the current frame towards setting a new physical model.
	ProcessForReference	Process the eye data from the current frame towards setting a new reference eye position.
	GetCalibrationUI	Gets the custom user interface of the calibration pipeline
